# Force measurement metrics for simulated elbow arthroscopy training

**DOI:** 10.1186/s40634-018-0157-1

**Published:** 2018-10-11

**Authors:** Nick F. J. Hilgersom, Tim Horeman-Franse, Ronald L. A. W. Bleys, Denise Eygendaal, Michel P. J. van den Bekerom, Gabriëlle J. M. Tuijthof, Elbow Study Collaborative, Bertram The, Carina L.E. Gerritsma, Lex Boerboom, Tom Roeling, Marco van der Pluijm, Michel P.J. van den Bekerom, Denise Eygendaal

**Affiliations:** 1 Department of Orthopaedic Surgery, Amsterdam University Medical Centres, University of Amsterdam, Amsterdam Movement Sciences, Meibergdreef 9, 1105 AZ Amsterdam, the Netherlands; 2 Department of Biomechanical Engineering, Delft University of Technology, Mekelweg 2, 2628 CD Delft, the Netherlands; 3 Department of Human Movement Sciences, Vrije Universiteit Amsterdam, De Boelelaan 1117, 1081 HV Amsterdam, the Netherlands; 4 Department of Anatomy, University Medical Centre Utrecht, Heidelberglaan 100, 3584 CX Utrecht, the Netherlands; 5 Department of Orthopaedic Surgery, Amphia Hospital, Molengracht 21, 4818 CK Breda, the Netherlands; 6 Department of Orthopaedic Surgery, Onze Lieve Vrouwe Gasthuis, Oosterpark 9, 1091 AC Amsterdam, the Netherlands; 7 Zuyd University of Applied Science, Nieuw Eyckholt 300, 6419 DJ Heerlen, the Netherlands

**Keywords:** Elbow, Arthroscopy, Navigational forces, Experts, Skills assessment, Education, Cadaver

## Abstract

**Background:**

Elbow arthroscopy is a difficult surgical technique. Objective metrics can be used to improve safe and effective training in elbow arthroscopy. Force exerted on the elbow tissue during arthroscopy can be a measure of safe tissue manipulation. The purpose of this study was to determine the force magnitude and force direction used by experts during arthroscopic elbow navigation in cadaveric specimens and assess their applicability in elbow arthroscopy training.

**Methods:**

Two cadaveric elbows were mounted on a Force Measurement Table (FMT) that allowed 3-dimensional measurements (x-, y-, and z-plane) of the forces exerted on the elbow. Five experts in elbow arthroscopy performed arthroscopic navigation once in each of two cadaveric elbows, navigating through the posterior, posterolateral and anterior compartment in a standardized fashion with visualization of three to four anatomic landmarks per compartment. The total absolute force (F_abs_) and force direction exerted (α and β) on the elbow during arthroscopy were recorded. α being the angle in the horizontal plane and β being the angle in the vertical plane. The 10th–90th percentiles of the data were used to set threshold levels for training.

**Results:**

The median F_abs_ was 24 N (19 N – 30 N), 27 N (20 N – 33 N) and 29 N (23 N – 32 N) for the posterior, posterolateral and anterior compartment, respectively. The median α was - 29° (- 55° – 5°), - 23° (- 56° – -1°) and 4° (- 22° – -18°) for the posterior, posterolateral and anterior compartment, respectively. The median β was - 71° (- 80° – -65°), - 76° (- 86° – -69°) and - 75° (- 81° – -71°) for the posterior, posterolateral and anterior compartment, respectively.

**Conclusion:**

Expert data on force magnitude and force direction exerted on the elbow during arthroscopic navigation in cadaveric specimens were collected. The proposed maximum allowable force of 30 N (smallest 90th percentile of F_abs_) exerted on the elbow tissue, and the 10th–90th percentile range of the force directions (α and β) for each compartment may be used to provide objective feedback during arthroscopic skills training.

## Background

Over the past decades elbow arthroscopy has become a surgical tool due to better understanding of the neurovascular anatomy, technical advancements, and broadening range of indications (Hilgersom et al., 2018; Yeoh et al., 2012). An increase in elbow arthroscopy use is expected to raise the number of complications, which emphasizes the importance of training in portal placement and arthroscopic skills to deliver safe surgical care (Rose & Pedowitz, 2015).

Arthroscopy requires excellent visual spatial awareness to mentally recreate a 3-dimenionsal environment from 2-dimensional images. This cannot be learned by assisting and observing in the operating theatre alone (Aggarwal et al., 2004; Aim et al., 2016; Rosenthal et al., 2006; Tashiro et al., 2009). Moreover, elbow arthroscopy specifically is technically challenging due to limited working space and close proximity of neurovascular structures (Hilgersom et al., 2017; Marshall et al., 1993; Miller et al., 1995; Omid et al., 2012; Stothers et al., 1995). Further distinguishing elbow arthroscopy is the need for mirrored hand-eye coordination in the lateral decubitus position when compared to most other arthroscopic modalities; and overhand versus underhand holding of instruments. All above, in combination with the lower frequency compared to knee or shoulder arthroscopy, makes it apparent that elbow arthroscopy has a longer learning curve in time.

Currently, no consensus exists on the minimal number of elbow arthroscopies that must be performed to become an expert. Savoie states that a minimal number of 100 performed elbow arthroscopies is necessary (Savoie 3rd, 2007). Furthermore, Claessen et al. (Claessen et al., 2017) observed a 30% complication rate in portal placement by novice surgeons, which was significantly higher compared to experienced elbow arthroscopists (Elfeddali et al., 2013; Marti et al., 2013). These numbers make clear that elbow arthroscopy (simulated) training is essential (Claessen et al., 2017; Rose & Pedowitz, 2015).

Cadaveric training is still the preferred training method to improve arthroscopic skills because it provides the most realistic setting (Camp et al., 2016; Hui et al., 2013; Koehler et al., 2015). Objective performance measurement by using metrics is preferred over global rating scales such as Objective Structured Assessment of Technical Skills (OSATS) (Horeman et al., 2016; Martin et al., 1997; van Hove et al., 2010). Such metrics have yet to be defined in elbow arthroscopy, but have been defined in knee and shoulder arthroscopy, for example to differentiate between levels of experience and to set thresholds for safe tissue manipulation (Stunt et al., 2014; Tashiro et al., 2009; Tuijthof et al., 2011). Recently, Obdeijn et al. (Obdeijn et al., 2016) defined a maximum allowable force magnitude of 7.3 N (90th percentile) using expert data derived thresholds and demonstrated that force direction is equally important as force magnitude for safe wrist arthroscopy to prevent cartilage damage. Similarly, forces exerted on the elbow by experts during elbow arthroscopy may also be valuable indicators of a safe elbow arthroscopy.

The purpose of this study was to determine the force magnitude and force direction used by experts during arthroscopic elbow navigation in cadaveric specimens and assess their applicability in elbow arthroscopy training.

## Methods

The study was designed to fit within the set time schedule of the two day-26th annual international Arthroscopy & Arthroplasty Courses Utrecht. This implied that we could perform data acquisition with five experts operating on two cadaveric specimens. This approach was suitable to meet the study goal, because a similar strategy was followed for assessing a threshold navigation force for wrist arthroscopy (Obdeijn et al., 2016): a) focus on experts and recruit as many as possible to determine if their navigation force variation is acceptably small to set a safety threshold; b) keep other conditions as constant as possible; and c) propose a safety margin (90th percentile) to cover for the effects of other conditions when setting the metrics’ threshold.

### Cadaveric specimen

Two fresh-frozen right-handed upper limb cadaver specimens without evidence of previous trauma, surgery or deformity were prepared to mimic an arthroscopic setting. These specimens were derived from bodies that entered the department of anatomy through a donation program. From these persons written consent was obtained during life that allowed the use of their entire bodies for educational and research purposes. Specimens were stored at − 20 °C and thawed 24 h before use. The upper limb cadaveric specimens arms were dissected transversely 15–20 cm proximal of the humeral epicondyles and mounted onto the custom-made static arm holder of the force measurement table (FMT) with the posterior humerus facing superiorly and the humeral epicondyles orientated horizontally, mimicking a lateral decubitus position (Horeman et al., 2016).

### Force measurement table

**Fig. 1:**
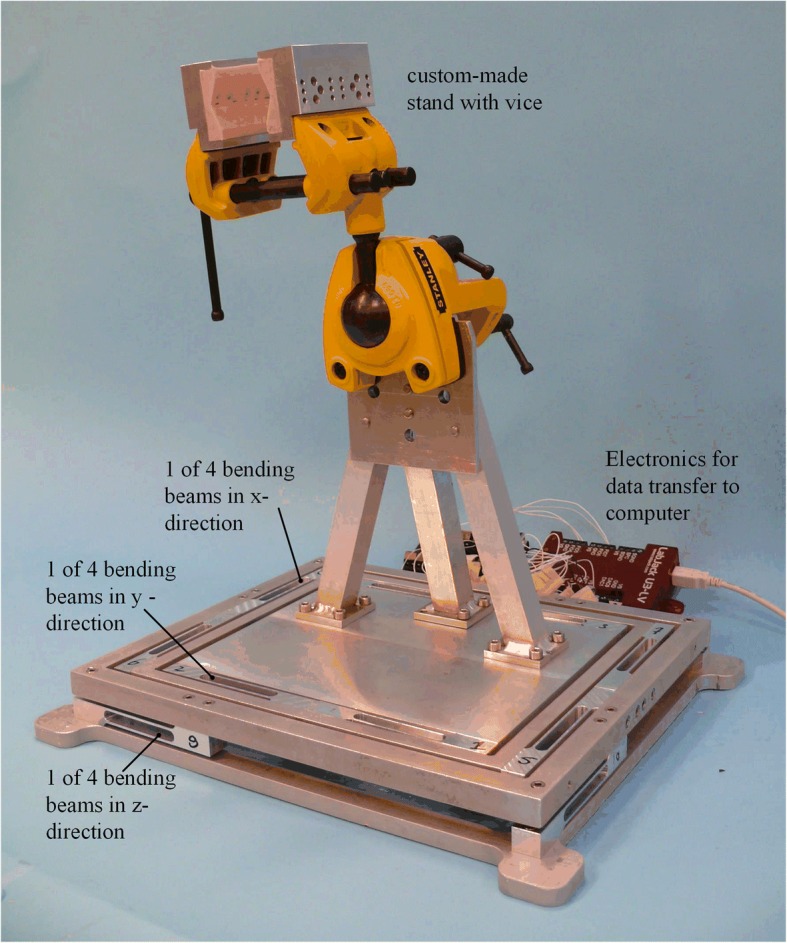
Force Measurement Table. The FMT with custom-made stand with vice attached is shown. The FMT consists of the three squared frames and bending beams with Hall effect sensors and magnets in the x-, y- and z-plane of the FMT. The design of the FMT allows continuous recordings of the forces exerted on the cadaver specimen attached to the vice in three loading directions

For the interested readers, the force measurement table is described in detail by Horeman et al. (Horeman et al., 2016). In short, when a cadaveric specimen is firmly fixated in the vice of the FMT, it measures the forces in x-, y-, z-direction during arthroscopic skills training, enabling objective performance tracking of the trainees. The FMT consists of three squared frames, each connected to one another by four beams that bend upon loading (Fig. 1). The three frames displace independently; each in a single direction (i.e. x-, y-, or z-direction) (Fig. 1). The applied force on each frame is calculated by measuring the relative displacement of the four bending beams and multiplication with the bending beams’ known stiffness (Fig. 1). Bending beam displacements were measured using Linear Hall effect sensors and Neodymium disk magnet built into the bending beams (Horeman et al., 2016). The FMT allowed continuous recording of the forces exerted on the cadaver elbow by the instruments in a range of 0 N to 750 N in three loading directions, with an accuracy of 0.1 N and a sample frequency of 24 Hz (Horeman et al., 2016). To position the elbow above the FMT, a custom-made stand with vice was mounted on the FMT. The vice allows fast mounting of the prepared humerus bone in a 45-degree angle to mimic the actual procedure (Fig. 1).

A camera tracking system using two digital video cameras set up on both sides of the operator and the arthroscope camera was set up for monitoring of instrument use, capture ‘occurrences’ (e.g.; probing of the predefined landmarks) and adequate postprocessing of the data acquired with the FMT.

Qualitative analyses of the individual contribution of the arthroscope and probe on the total forces exerted on the cadaver elbow was performed by combining the data from the FMT and camera tracking system.

### Experts

The expert group consisted of five upper limb surgeons specialized in elbow arthroscopy and instructors at the 26th annual international Arthroscopy & Arthroplasty Courses Utrecht. The experts filled out a questionnaire to document their demographic data (Table 1).

**Table 1:** Demographic data and experience of the five participants

Expert	1	2	3	4	5
Age (years)	42	38	44	50	48
Gender	Male	Male	Male	Female	Female
Dexterity	Right	Right	Left	Right	Right
Expertise	Expert	Expert	Expert	Expert	Expert
Exp EA (years)	2	4	8	16	15
NR EA (year)	100	100	20–25	100	10–15

*Exp* Experience*,* EA *Elbow arthroscopy*, NR *Number*

Prior to the experiment, expert one created the following arthroscopic portals in both cadaveric specimens; proximal anteromedial, proximal anterolateral, midtricipital, posterolateral and soft spot portal, and as routinely is performed with elbow arthroscopy, shaved fibrous tissue blocking the view. The midtricipital, posterolateral and proximal anteromedial portal served as viewing portals for the posterior, posterolateral and anterior compartment, respectively.

**Fig. 2:**
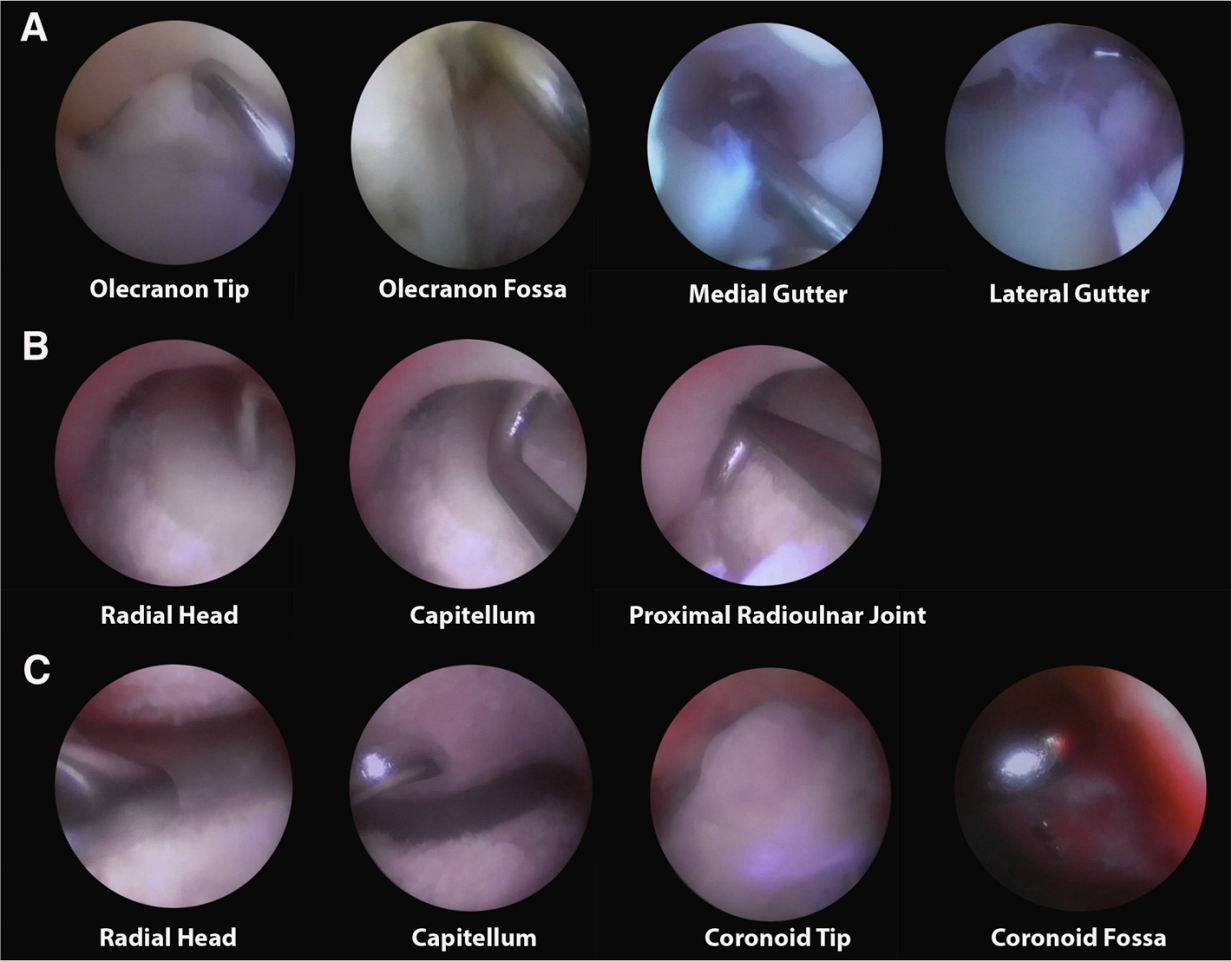
Arthroscopic views of predefined landmarks per compartment. **a** Posterior compartment. **b** Posterolateral compartment. **c** Anterior compartment

Each expert performed an arthroscopic navigation once on each cadaveric elbow using the above-described portals. During the arthroscopic navigation experts consecutively visualized the posterior, posterolateral and anterior compartment and were asked to determine the predefined landmarks (Fig. 2). In the posterior compartment the landmarks were the olecranon tip, olecranon fossa, medial gutter, and lateral gutter (Fig. 2a). In the posterolateral compartment the landmarks were the radial head, capitellum, and proximal radioulnar joint (Fig. 2b). In the anterior compartment the landmarks were the radial head, capitellum, coronoid tip, and coronoid fossa (Fig. 2c). Each landmark had to be touched by the probe and visualised in the centre of the arthroscopic image. Once a landmark was visualized per protocol, as visually verified by one of the researchers, the expert could proceed to the next anatomic landmark. Arthroscopic elbow navigation was performed in the same consecutive order of experts on both elbow specimens. All measurements were performed on the same day. During the experiment experts could extend the elbow as they felt necessary for proper portal placement and instrument use. All arthroscopic tasks were performed using an arthroscopic probe and a 30°-angle 4 mm arthroscope from Karl Storz (Tuttlingen, Germany).

The experts were asked to perform the tasks as they would be performing live surgery on an actual patient.

### Data processing

The data gathered with the FMT and camera tracking system were processed using *Matlab* (version R2014a, The Mathworks, Natick, MA, USA) and *IBM SPSS statistics* (version 22, SPSS, Chicago, IL, USA). All raw voltage data were filtered with a low-pass Butterworth filter with a cut-off frequency of 24 Hz to suppress high-frequency noise. For each compartment the total absolute force (F_abs_) per sample was calculated by summation of the force measurements in the x-, y-, and z-plane after the force measurement in z-direction was compensated for the mass of the specimen and holder. In addition, the force direction in the horizontal plane (α) could be derived from the force magnitude in the x- and y-plane, and the force direction in the vertical plane (β), which is aligned with the humerus mounted on the set-up, from the force magnitude in the x- and z-plane. A positive α-angle implies a direction of force to the lateral side and a negative α-angle implies direction of force to the medial side. A positive β-angle implies upward direction of force and a negative β-angle implies downward direction of force.

### Statistical analysis

The presence of normal distributions for F_abs_, α and β was determined with the Kolmogorov-Smirnov test per compartment. As the data were not normally distributed, F_abs_, α and β were expressed in terms of median (10th–90th percentile). A Mann-Whitney U-test was performed to compare the F_abs_ measurements for the anterior compartment between the two cadaveric specimens (*p* < 0.05).

Prior to this study, Obdeijn et al. (Obdeijn et al., 2016) successfully applied the 10th and 90th percentiles to set thresholds for safe tissue manipulation and force direction in wrist arthroscopy. Therefore, we used a similar strategy in this study: the 10th and 90th percentiles of F_abs,_ α and β were used to set threshold levels for safe tissue manipulation and force direction that can be used during elbow arthroscopy training.

## Results

**Fig. 3:**
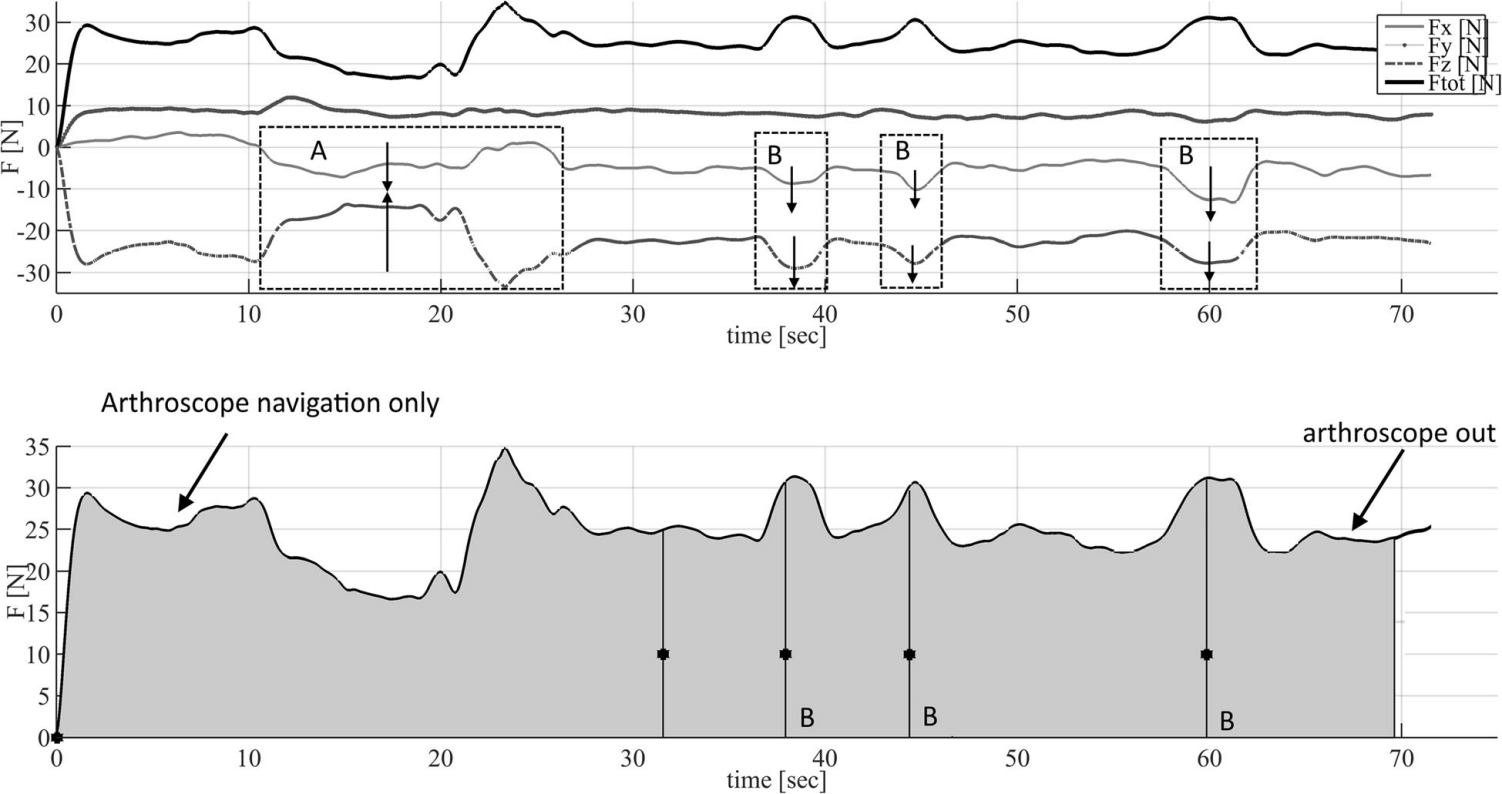
Example of force measurement in time of one navigation task performed by one expert. This example shows force measurement in time of one navigation task performed by one expert in the posterior compartment. In the upper graph the individual force components as well as the overall combined force F_abs_ are illustrated. In the lower graph the stars indicate the moments of touching and displaying the assigned landmark. In this 2D representation, the first Area (A) represents a location were elbow flexing occurs indicated by an oppositely directed change in Force. The following area’s (B) represent instrument bone/tissue interaction with force fluctuations in similar direction

Figure 3 shows an example of the force measurement in time of one navigation task performed by one expert in the posterior compartment. A qualitative initial analysis combining the force data and video footage showed force fluctuations in a similar direction during probing of a landmark, force fluctuations in an opposite direction during elbow flexion, and only marginal variation in forces during instrument changes when only the arthroscope was in place (Fig. 3).

**Fig. 4:**
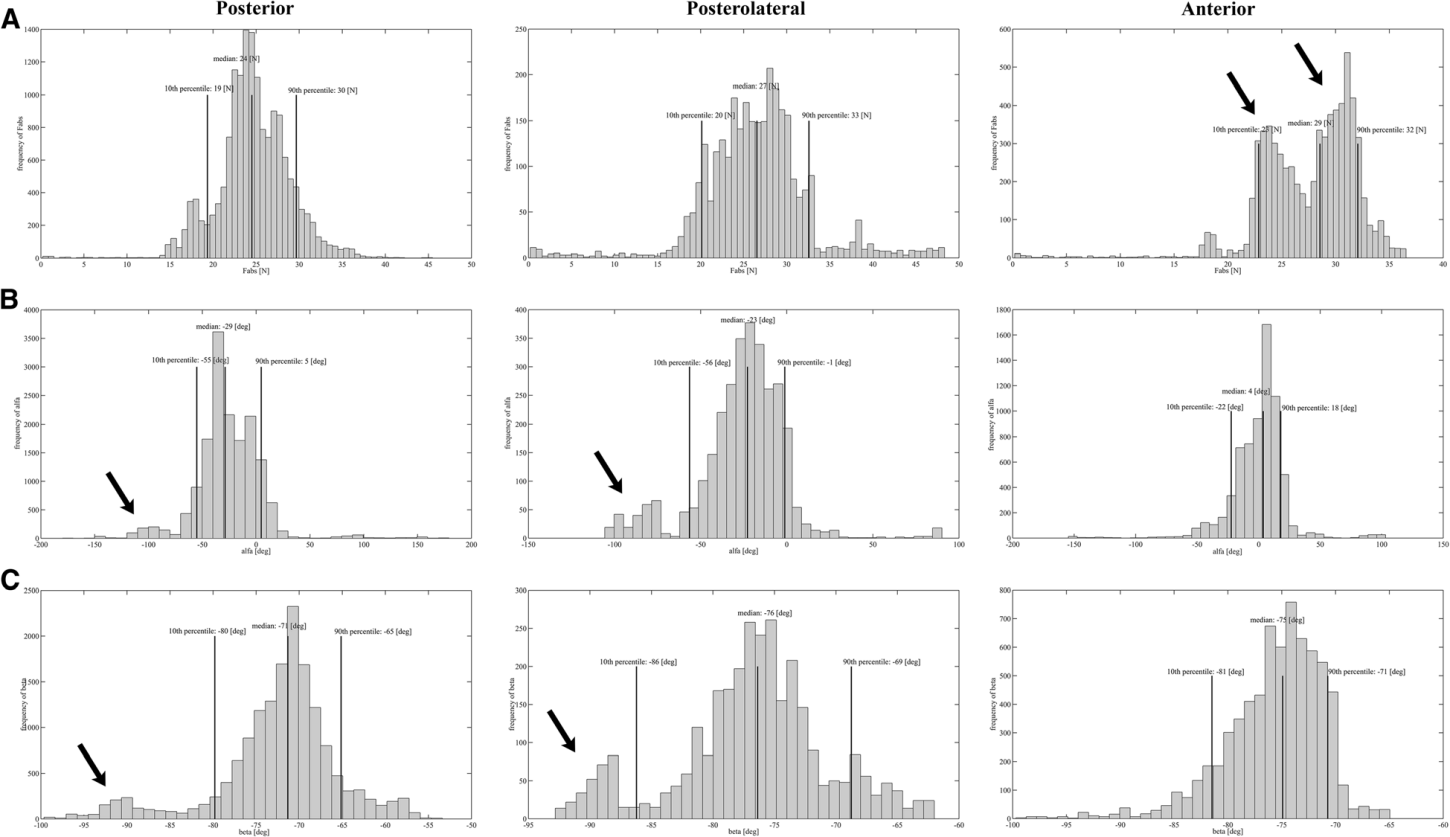
Histograms presenting the data points for F_abs_, alfa and beta of each compartment. Histograms presenting the median, 10th and 90th percentile for frequency of total absolute force (F_abs_), horizontal angle (α) and vertical angle (β) data points in the posterior, posterolateral and anterior compartment of both cadaver elbows. **a** Histogram showing F_abs_. The two black arrows point to two separate peaks in frequency of F_abs_, around 23 N and 30 N in the anterior compartment. **b** Histogram showing α. α is positive to the right, and negative to the left. The two black arrows point to a smaller peak around − 90° for α and β in the posterior and posterolateral compartment. **c** Histogram showing β. β is positive upward, and negative downward. The two black arrows point to a smaller peak around − 90° for α and β in the posterior and posterolateral compartment

The histograms of F_abs_, α and β for the posterior, posterolateral and anterior compartment of both cadaveric elbows are presented in Fig. 4.

### Total absolute of force F_abs_

The median F_abs_ is similar for each compartment, being 24 N (range 19 N – 30 N) for the posterior compartment, 27 N (20 N – 33 N) for the posterolateral compartment, and 29 N (23 N – 32 N) for the anterior compartment (Fig. 4a). In the anterior compartment, two peaks of F_abs_ are observed in the histogram, one around 23 N of absolute force and one around 30 N of absolute force (Fig. 4a). The Mann-Whitney U-test indicated a significant difference between the values of F_abs_ for the anterior compartment between the two cadaveric specimens (*p* < 0.05).

### Horizontal angle (α) and vertical angle (β)

**Fig. 5:**
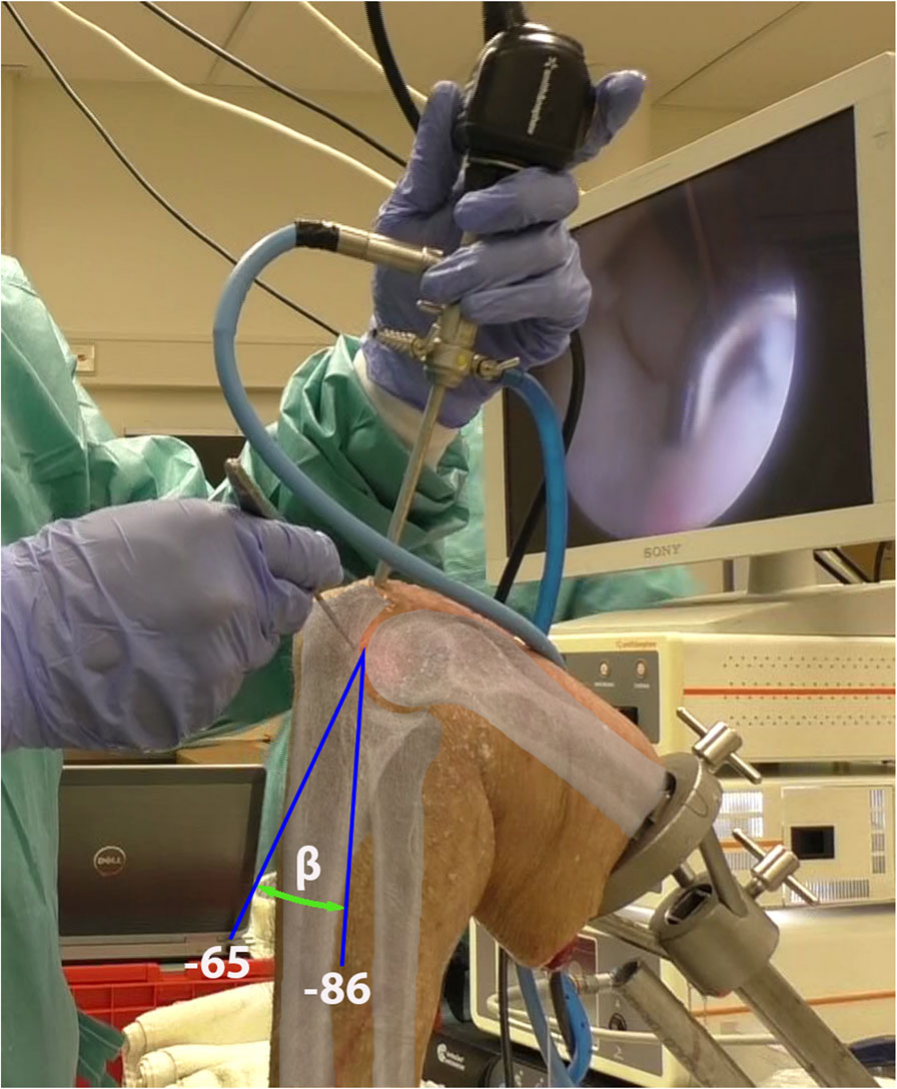
Median force direction in the vertical plane (β) for all compartments combined. Schematic representation of the median direction of Fabs in the vertical plane for all compartments combined represented by β. The blue lines represent the lowest and highest values of the 10th and 90th percentiles of β. An elbow x-ray has been superimposed over the cadaveric elbow to further clarify the correlation between the overall median force direction and elbow joint

**Fig. 6:**
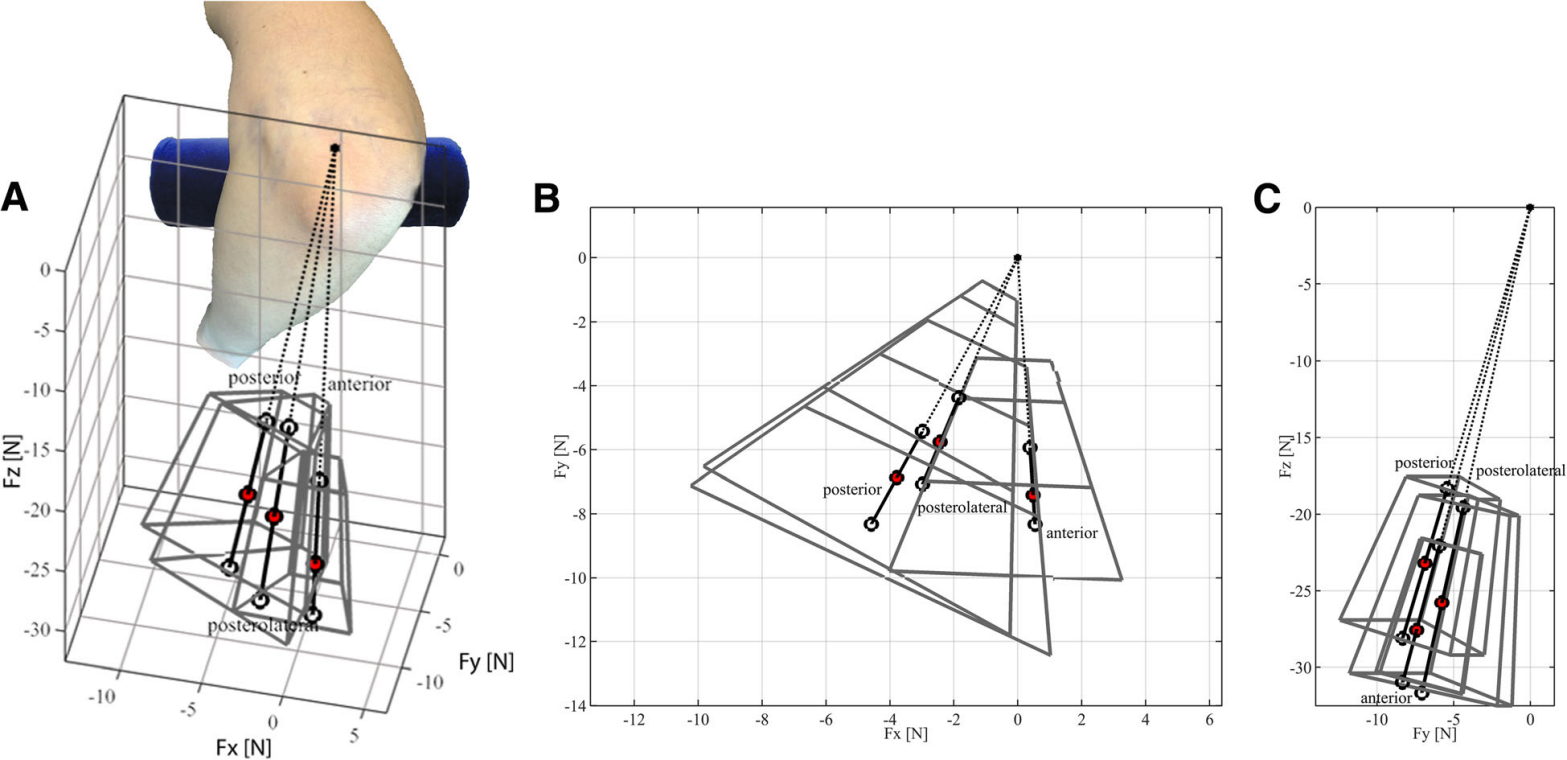
Safe force zone (magnitude and direction) for all compartments. The red dots indicate the median value of F_abs_, α and β for each compartment. The thick black lines with white dots at their respective ends represent the 10th and 90th percentile value of F_abs_ in the median force direction. The grey boxes surrounding the thick black lines indicate the combined boundaries of the 10th -90th percentiles for F_abs_, α and β. The origin is taken at the same position for each compartment. **a** 3D graphical representation. For reference an elbow in the lateral decubitus position is added. So the forces are directed towards the surgeon, **b** Top view, **c** Sagittal view

The median α, force direction in the horizontal plane, is − 29° for the posterior compartment with a range of 60°, is − 23° for the posterolateral compartment with a range of 55° and is 4° for the anterior compartment with a range of 40° (Fig. 4b). Notable is the more medial direction and smaller range of α in the anterior compartment compared to the posterior and posterolateral compartment (Figs.4b and 6b). The median β, force direction in the vertical plane, is − 71° for the posterior compartment with a range of 15°, − 76° for the posterolateral compartment with a range of 17° and − 75° for the anterior compartment with a range of 10° (Fig. 4c). The median β remains fairly constant with a maximum difference of 5° and maximum range of 17° (Fig. 6c). Figure 5 provides a schematic representation of the median β for all compartments combined.

Comparison of the force direction between the different compartments showed a second smaller peak around − 90 degrees for α and β in the posterior and posterolateral compartment (Fig. 4b and c). Expert 1, who created the portals, had a substantial share in this peak, particularly when performing the task in the first cadaveric specimen.

### Safe zone – Metric threshold

Finally, a graphical interpretation is given in Fig. 6 of the median values of the F_abs_, α and β for each of the three compartments (posterior, posterolateral and anterior) as well as the set safe zone using the 10th and 90th percentile force values from Fig. 4*.* The 90th percentile values indicate the set maximum threshold for the metric.

## Discussion

This study shows that median loads of 24-29 N are exerted on the elbow by experts during arthroscopic navigation in a cadaveric elbow. These loads represent the combined forces exerted by the arthroscope and the probe on the anatomic structures of the elbow. The overall measured forces are considerably higher than expert force data for wrist arthroscopy (median F_abs_ of 3.8 N) (Obdeijn et al., 2016) and probing of meniscal tissue in the knee (mean F_abs_ ranging 2.8–3.9 N) (Tuijthof et al., 2011), but they are lower than expert force data found for knee joint distraction (mean F_abs_ of 43-50 N) (Stunt et al., 2014).

A possible contributing factor to the overall higher force load is that manoeuvring the arthroscope to a compartment is performed primarily by knowing the correct orientation of the arthroscope and by haptic feedback using the bony structures for guidance, such as sliding along the anterior face of the humerus to create the proximal anteromedial portal, or using bony structures as a support point/wedge to take a corner while navigating around the elbow (Fig. 5). In addition to a lack of joint distraction, this relative high bone-instrument loading may cause a higher overall loading on the elbow. The consequences of the relative high force may be limited, because surgical procedures during elbow arthroscopy are primarily performed outside of the articulating surfaces of the elbow joint (i,e. synovectomy, capsular release, loose body removal). This reduces the chance of injury to delicate tissues inside the joint such as the poorly healing articular cartilage.

The arthroscope assembly (e.g. arthroscope, cables, camera) and supporting hand plus arm most likely have the highest contribution in the total force as combined analysis of video footage and force data with the aim to correlate force direction and variation in force magnitude to instrument use showed hardly any variation in forces during probing of landmarks or instrument changes with the arthroscope in place (Figs. 3 and 5). This is a possible assumption as the FMT measured the total combined forces exerted on the cadaveric elbow and is unable to quantitatively assess the individual contribution of the arthroscope or probe used during elbow arthroscopy.

Based on expert data, the 10th and 90th percentiles of the exerted force have been used to determine force thresholds in wrist arthroscopy and probing of menisci (Obdeijn et al., 2016; Tuijthof et al., 2011). Utilizing the same strategy on current expert data, we propose a maximum allowable force load of 30 N to be exerted on the elbow during arthroscopic navigation, which is the smallest value of 90th percentiles of the force magnitude of all three compartments (Fig. 4a). This threshold level should be demonstrated in elbow arthroscopy training to let novices experience the feel of the magnitude of a load around 30 N, as this is a most likely a lot higher than novices expect (Obdeijn et al., 2014; Tuijthof et al., 2011). This can help students to train their haptic senses in a safe way by preventing them to use higher loads.

The median force direction and range during arthroscopic navigation in the elbow is similar for all compartments in the vertical plane (β) (Figs. 4c, 5 and 6c). The median force direction of the anterior compartment in the horizontal plane (α) is more medial compared to the posterior and posterolateral compartment, and the range of force direction is smaller when compared to the posterior and posterolateral compartment (Fig. 4b and 6b). These findings can be related to working through the proximal anteromedial portal, the anatomical location of the anterior compartment and the anatomical distance between the landmarks in the anterior compartment, respectively.

The second smaller peak in force direction in both planes (α and β) observed around − 90° in the posterior and posterolateral compartment (Fig. 4b and c) seems attributable to suboptimal portal placement as expert 1 who created the portals had a substantial share in this second peak, particularly in the first specimen. This is supported by the lower median F_abs_ used by expert 1 in cadaver 1. Expert 1 created the portals and as such knew the exact orientation of the portals resulting in a lower median F_abs_ compared to the other experts.

Elbow arthroscopy, when performed with the patient in a lateral decubitus position, requires a mirrored way of instrument handling with a 30° arthroscope when compared to performing arthroscopy of most other joints. The force direction in the vertical plane (β) shows minor variation (Figs. 5 and 6c), which is a sign that this range may be used for novice surgeons to strive for. The latter is strengthened by Obdeijn et al. (Obdeijn et al., 2016; Obdeijn et al., 2014) who showed that force direction is equally as important as force magnitude, and found that novices showed considerable variation in loading direction compared to experts when performing wrist arthroscopy. The force direction area defined by the 10th–90th percentile of expert thresholds for α and β (Figs. 5 and 6) may be used to adjust the direction of the arthroscope to properly navigate through the complex elbow anatomy. To be of assistance for the trainees, it is necessary to visualize the direction of force on the video screen via augmented reality. Implementing this in a meaningful way is a challenging task, as is shown by the work of Smit et al. (Smit et al., 2017).

There are limitations to this study. First, although the number of data points per surgeon was high, the number of experts and cadavers was small, but feasible within the set time frame of the advanced elbow course. Besides the variation amongst the experts, other conditions (cadavers, the joint status in time and portal placement) do effect the forces. Since our aim was not to assess the individual contribution of each condition, but rather set an overall safety threshold, we argue that the small group of surgeons conducting the trials on two cadavers should represent the entire group of expert elbow arthroscopists sufficiently. This is supported by the narrow range of the 10th–90th percentile of the median values of F_abs_, α and β. Second, the data was collected from cadaveric specimens that are usually stiffer than elbows from live patients. Therefore, one can reason that higher forces will be observed when performing arthroscopy on living patients. However, this may be partly compensated as cadaveric specimen are commonly obtained from elderly people with usually lesser tissue quality than young people.

Although cadaveric training provides the most realistic experience, cadaveric training is not the preferred method to start training elbow arthroscopy skills. First, because cadaveric training is expensive and there is limited availability (Camp et al., 2016; Stirling et al., 2014). Moreover, as was also shown in this study, the anatomic variation amongst cadaveric specimen as well as their joint status in due to time compromises similar training conditions for a certain amount of repetitions or trainees. For example, in the present study two peaks of F_abs_ were observed during navigation of the anterior compartment, around 23 N and 30 N (Fig. 4a), which were attributable to the use of two cadaver elbows (Mann-Whitney U, *p* < 0.05). In addition, due to continuous water irrigation of the elbow for a long duration (five elbow arthroscopies) the soft tissues would swell, possibly making portal insertion, gaining orientation and working inside the joint more difficult. In this study, this was observed as moderate differences in the median F_abs_ of 8.2 N and 3.5 N between the first and last expert in the first and second cadaver elbow, respectively. Consequently, threshold levels as determined in this study should be adjusted per cadaver and training time on the cadaver (swelling due to irrigation). Therefore, we recommend starting training basic elbow arthroscopic skills on a simulator. This will provide the same standard for all trainees at any time, and allows adequate objective feedback by setting one threshold value and facilitates observation of training progress of participants compared to their peers. After obtaining proficiency in basic arthroscopic skills on a simulator, a trainee may advance to cadaveric skills training to become acquainted with the feeling and effect of the loads on human tissues along with learning to adapt to anatomic variations as is the case in live surgery.

Nonetheless, this study shows that force data can be accurately and reliably recorded in three loading directions using the FMT (Horeman et al., 2016), allowing expert thresholds to be defined for force magnitude and force directions that can be used for objective feedback during elbow arthroscopy training.

## Conclusions

Expert data on force magnitude and force direction exerted on the elbow during arthroscopic navigation in cadaveric specimens was collected. The proposed maximum allowable force of 30 N (smallest 90th percentile of F_abs_) exerted on the elbow tissue, and the 10th–90th percentile range of the force directions (α and β) for each compartment may be used to provide objective feedback during arthroscopic skills training.

## Abbreviations

F_abs_: Total absolute force
FMT: Force Measurement Table
OSATS: Objective Structured Assessment of Technical Skills

## References

[CR1] Aggarwal R, Moorthy K, Darzi A (2004). Laparoscopic skills training and assessment. Br J Surg.

[CR2] Aim F, Lonjon G, Hannouche D, Nizard R (2016). Effectiveness of virtual reality training in Orthopaedic surgery. Arthroscopy.

[CR3] Camp CL, Krych AJ, Stuart MJ, Regnier TD, Mills KM, Turner NS (2016). Improving resident performance in knee arthroscopy: a prospective value assessment of simulators and cadaveric skills laboratories. J Bone Joint Surg Am.

[CR4] Claessen F, Kachooei AR, Kolovich GP, Buijze GA, Oh LS, van den Bekerom MPJ, Doornberg JN (2017). Portal placement in elbow arthroscopy by novice surgeons: cadaver study. Knee Surg Sports Traumatol Arthrosc.

[CR5] Elfeddali R, Schreuder MH, Eygendaal D (2013). Arthroscopic elbow surgery, is it safe?. J Shoulder Elb Surg.

[CR6] Hilgersom NF, Oh LS, Flipsen M, Eygendaal D, van den Bekerom MP (2017). Tips to avoid nerve injury in elbow arthroscopy. World J Orthop.

[CR7] Hilgersom NFJ, Molenaars RJ, van den Bekerom MPJ, Eygendaal D, Doornberg JN (2018). Review of Poehling et al (1989) on elbow arthroscopy: a new technique. J ISAKOS.

[CR8] Horeman T, Tuijthof GJM, Wulms PB, Kerkhoffs GMMJ, Gerards RM, Karahan M (2016). A force measurement system for training of arthroscopic tissue manipulation skills on cadaveric specimen. J Med Devices.

[CR9] Hui Y, Safir O, Dubrowski A, Carnahan H (2013). What skills should simulation training in arthroscopy teach residents? A focus on resident input. Int J Comput Assist Radiol Surg.

[CR10] Koehler R, John T, Lawler J, Moorman C, Nicandri G (2015). Arthroscopic training resources in orthopedic resident education. J Knee Surg.

[CR11] Marshall PD, Fairclough JA, Johnson SR, Evans EJ (1993). Avoiding nerve damage during elbow arthroscopy. J Bone Joint Surg Br.

[CR12] Marti D, Spross C, Jost B (2013). The first 100 elbow arthroscopies of one surgeon: analysis of complications. J Shoulder Elb Surg.

[CR13] Martin JA, Regehr G, Reznick R, MacRae H, Murnaghan J, Hutchison C, Brown M (1997). Objective structured assessment of technical skill (OSATS) for surgical residents. Br J Surg.

[CR14] Miller CD, Jobe CM, Wright MH (1995). Neuroanatomy in elbow arthroscopy. J Shoulder Elb Surg.

[CR15] Obdeijn MC, van Baalen SJ, Horeman T, Liverneaux P, Tuijthof GJ (2014). The use of navigation forces for assessment of wrist arthroscopy skills level. J Wrist Surg.

[CR16] Obdeijn MC, Horeman T, de Boer LL, van Baalen SJ, Liverneaux P, Tuijthof GJ (2016). Navigation forces during wrist arthroscopy: assessment of expert levels. Knee Surg Sports Traumatol Arthrosc.

[CR17] Omid R, Hamid N, Keener JD, Galatz LM, Yamaguchi K (2012). Relation of the radial nerve to the anterior capsule of the elbow: anatomy with correlation to arthroscopy. Arthroscopy.

[CR18] Rose K, Pedowitz R (2015). Fundamental arthroscopic skill differentiation with virtual reality simulation. Arthroscopy.

[CR19] Rosenthal R, Gantert WA, Scheidegger D, Oertli D (2006). Can skills assessment on a virtual reality trainer predict a surgical trainee's talent in laparoscopic surgery?. Surg Endosc.

[CR20] Savoie FH (2007). Guidelines to becoming an expert elbow arthroscopist. Arthroscopy.

[CR21] Smit D, Spruit E, Dankelman J, Tuijthof G, Hamming J, Horeman T (2017). Improving training of laparoscopic tissue manipulation skills using various visual force feedback types. Surg Endosc.

[CR22] Stirling ER, Lewis TL, Ferran NA (2014). Surgical skills simulation in trauma and orthopaedic training. J Orthop Surg Res.

[CR23] Stothers K, Day B, Regan WR (1995). Arthroscopy of the elbow: anatomy, portal sites, and a description of the proximal lateral portal. Arthroscopy.

[CR24] Stunt JJ, Wulms PH, Kerkhoffs GM, Sierevelt IN, Schafroth MU, Tuijthof GJ (2014). Variation in joint stressing magnitudes during knee arthroscopy. Knee Surg Sports Traumatol Arthrosc.

[CR25] Tashiro Y, Miura H, Nakanishi Y, Okazaki K, Iwamoto Y (2009). Evaluation of skills in arthroscopic training based on trajectory and force data. Clin Orthop Relat Res.

[CR26] Tuijthof GJ, Horeman T, Schafroth MU, Blankevoort L, Kerkhoffs GM (2011). Probing forces of menisci: what levels are safe for arthroscopic surgery. Knee Surg Sports Traumatol Arthrosc.

[CR27] van Hove PD, Tuijthof GJ, Verdaasdonk EG, Stassen LP, Dankelman J (2010). Objective assessment of technical surgical skills. Br J Surg.

[CR28] Yeoh KM, King GJ, Faber KJ, Glazebrook MA, Athwal GS (2012). Evidence-based indications for elbow arthroscopy. Arthroscopy.

